# Efficacy of Repetitive Transcranial Magnetic Stimulation on Postoperative Delirium in Elderly Patients Undergoing Non‐Cardiac Major Surgery: A Randomized Controlled Trial

**DOI:** 10.1002/brb3.71242

**Published:** 2026-01-30

**Authors:** Wen‐Bo Gao, Wen‐Hui Wang, Si‐Tong Zhou, Zi‐Wei Lu, Xin Xiang, Jin Hu, Ting‐Yu Jin, Chao‐Bo Ni, Ming Yao, Hua‐Dong Ni

**Affiliations:** ^1^ Department of Anesthesia Medicine Zhejiang Chinese Medical University Hangzhou Zhejiang China; ^2^ Department of Neurology The Affiliated Hospital of Jiaxing University Jiaxing Zhejiang China; ^3^ Department of Anesthesiology and Pain Medicine The Affiliated Hospital of Jiaxing University Jiaxing Zhejiang China

**Keywords:** aged, non‐cardiac surgery, postoperative delirium, repetitive transcranial magnetic stimulation

## Abstract

**Background:**

Postoperative delirium (POD) is a frequent complication among elderly surgical patients and is associated with adverse outcomes and increased mortality. Current preventive and therapeutic strategies remain limited. Repetitive transcranial magnetic stimulation (rTMS) has recently shown promise in enhancing cognitive function across various neurological and psychiatric conditions.

**Objective:**

This trial aimed to investigate the efficacy of preoperative rTMS on POD in elderly patients undergoing elective non‐cardiac surgery.

**Methods:**

This double‐blind, randomized controlled trial included 254 patients aged 60 years or older undergoing elective non‐cardiac surgery, randomly assigned to either active rTMS group or sham rTMS group. Patients received two sessions of 10 Hz rTMS over the left DLPFC, at 110% RMT, totaling 1080 pulses before surgery. The primary outcome was the incidence of POD within 7 days after surgery.

**Results:**

In the intention‐to‐treat analysis of 249 patients (median age 69 years [IQR 63 to 73] years; 46.2% women), the incidence of POD was significantly lower in the active rTMS group (10 of 124 [8.1%]) compared with the sham rTMS group (36 of 125 [28.8%]) (relative risk, 0.22; 95% CI 0.10 to 0.46; *p* < 0.001). Compared with the sham rTMS group, patients in the active rTMS group had significantly lower pain intensity and sleep quality on postoperative days 1 and 3 (*p* < 0.001 for each), lower anxiety and depression scores on postoperative days 3 and 7 (*p* < 0.001 for each), and lower frailty scores on postoperative days 1 and 7 (*p* < 0.001 for each), while there was no significant differences in PONV scores at any time pointy (*p* > 0.05 for each).

**Conclusions:**

Preoperative high‐frequency rTMS targeting left DLPFC was associated with a reduced incidence of POD in elderly patients undergoing elective non‐cardiac surgery.

## Graphical abstract

In this double‐blind randomized controlled trial, preoperative 10‐Hz rTMS targeting the left DLPFC reduced postoperative delirium within 7 days in older adults undergoing elective non‐cardiac surgery compared with sham stimulation and was well tolerated.

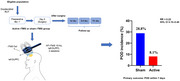

**Abbreviations**
AD: Alzheimer's disease
aORs: Adjusted odds ratios
ASA: American Society of Anesthesiologists
BBB: Blood–brain barrier
CAM: Confusion Assessment Method
CAM‐ICU: Confusion Assessment Method for Intensive Care Unit
CIs: Confidence intervals
DLPFC: Dorsolateral prefrontal cortex
DMN: Default mode network
DRS‐R‐98: Delirium Rating Scale Revised‐98
FPN: Frontoparietal control network
HADS: Hospital Anxiety and Depression Scale
ITT: Intention‐to‐treat
MCI: Mild cognitive impairment
MEPs: Motor evoked potentials
MMSE: Mini‐Mental State Examination
NRS: Numeric Rating Scale
PND: Perioperative neurocognitive disorders
POD: Postoperative delirium
PONV: Postoperative nausea and vomiting
PP: Per‐protocol
PSCI: Post‐stroke cognitive dysfunction
RR: Relative risk
rTMS: Repetitive transcranial magnetic stimulation
SDRS: Sleep Dysfunction Rating Scale
VIF: Variance inflation factors

## 1 Introduction

Postoperative delirium (POD) is an acute neuropsychiatric syndrome of the central nervous system, characterized by inattention, altered levels of consciousness, and acute disturbances in cognitive function, mostly occurring the first postoperative day to one week after surgery (Evered et al. 2018; Jin et al. 2020). In older surgical patients, the incidence of POD is ranges from 5% to 50% (Daiello et al. 2019; Gleason et al. 2015). POD is one of the common postoperative complications in elderly patients, and it can adversely affect prognosis, prolong hospitalization, and increase the risk of mortality and dementia incidence (Gleason et al. 2015; Kunicki et al. 2023; Zhang et al. 2020). Multiple risk factors contribute to POD, with the most significant being old age, an American Society of Anesthesiologists (ASA) physical status > 2, Charlson Comorbidity Index (CCI) ≥ 2, and lower Mini‐Mental State Examination (MMSE) score (Aldecoa et al. 2024; Mevorach et al. 2023). The pathophysiological mechanisms of POD are multifactorial and commonly involve neuroinflammation, neuronal aging, neurotransmitter imbalances, and disrupted brain network connectivity (Maldonado 2013; Wilson et al. 2020). These risk factors and mechanisms do not act independently; they interact synergistically to promote the onset and progression of POD. Given this complexity, prevention has become a clinical priority. The ASA has proposed six strategies to reduce the incidence of perioperative neurocognitive disorders (PND): multidisciplinary education and training, cognitive assessment, delirium screening, non‐pharmacological interventions, pain management, and avoidance of antipsychotics and anxiolytics (Peden et al. 2021). Among these, non‐pharmacological interventions are considered the most effective (Peden et al. 2021; Qureshi and Arthur 2023; Swarbrick and Partridge 2022). As the geriatric population grows (World Health Organization Ageing and Health 2020; Yan et al. 2023), the number of elderly patients undergoing surgical procedures is also rising. Therefore, there is an urgent need for evidence‐based perioperative strategies to reduce the incidence of POD.

Repetitive transcranial magnetic stimulation (rTMS) is a non‐invasive brain stimulation technique that activates the cerebral cortex through electric current induced by rapidly changing magnetic field, thereby influencing local and distal neuronal activity, inducing neuroplasticity, and modulating cortical excitability (Klomjai et al. 2015). rTMS exhibits frequency‐dependent effects: low‐frequency rTMS (LF‐rTMS≤1 Hz) generally inhibits cortical excitability, whereas high‐frequency rTMS (HF‐rTMS >1 Hz) enhances it (Klomjai et al. 2015). Current evidence indicated that rTMS modulates cognition through multiple mechanisms, including regulation of cortical excitatory, neuroplastic reorganization, alterations in neurotransmitter dynamics, and enhancement network connectivity (Jiang et al. 2020; Klomjai et al. 2015). Owing to non‐invasive, safe, and minimal side effects, rTMS has been demonstrated to be effective in treating a broad spectrum of neurological and psychiatric disorders (Lefaucheur et al. 2020; Sanches et al. 2020). Studies had shown that rTMS can improve cognitive function in patients with mild cognitive impairment (MCI), Alzheimer's disease (AD), and Alzheimer's disease‐related dementias (Pagali et al. 2024).

The dorsolateral prefrontal cortex (DLPFC), a key region of the frontal cortex, plays a central role in attention, memory, and emotional regulation. It is also a core component of the frontoparietal control network (FPN), which is crucial for flexible cognitive function, executive control, and coordination with other important brain networks (Dengler et al. 2024). These features make the DLPFC a widely used target for rTMS. However, evidence on whether perioperative rTMS targeting the DLPFC can prevent postoperative delirium remains limited. One randomized trial in older adults undergoing major abdominal surgery reported that active rTMS delivered after extubation over the left DLPFC was associated with a lower incidence of POD (Zhou et al. 2025).

However, whether rTMS administered at different perioperative time points can prevent delirium across broader surgical populations remains unclear. Therefore, this randomized controlled trial applied HF‐rTMS (10 Hz) to the left DLPFC in the preoperative period to investigate its effect on the incidence of POD in elderly patients undergoing non‐cardiac surgery.

## 2 Material and Methods

### 2.1 Study Design

This single‐center, prospective, double‐blind, randomized controlled clinical trial was conducted at the Affiliated Hospital of Jiaxing University from December 2024 to August 2025. The trial was approved by the Ethics Committee of the Affiliated Hospital of Jiaxing University in September 2024 (Ethics identifier: 2024‐LY‐794) and completed registration at the China Clinical Trial Registry in December 2024 (Identifier: CHiCTR2400094439). Written informed consent from all participants or their legal representatives has been obtained prior to randomization. The study followed theConsolidated Standards of Reporting Trials (CONSORT) guidelines for reporting randomized clinical trials.

### 2.2 Participants

Participants aged 60 years or older, ASA physical status class ≤ III, and undergoing elective non‐cardiac surgery under general anesthesia with anesthesia time ≥ 2 h were eligible for trial inclusion. A total of 358 participants were screened. The exclusion criteria were as follows: (1) the participants and their families refuse to sign the consent form; (2) preoperative cognitive impairment, defined as an MMSE score below education‐adjusted cutoffs (≤17 for illiterate, ≤20 for primary school, ≤22 for middle school, and ≤23 for high school/college), based on previously validated normative and epidemiological evidence accounting for the influence of education (Crum et al. 1993; Jia et al. 2020; Zhang et al. 1990); (3) metal implants in the body; (4) current or previous history of neurological or psychiatric disorders; (5) cranial disease, or history of previous cranial surgery; (6) severe cardiovascular and cerebrovascular diseases, liver and kidney insufficiency or other serious system diseases; (7) neurosurgery; (8) long‐term use of sedatives, psychotropic drugs, opioids, history of drug dependence, and history of alcoholism; and (9) those who are allergic to anesthetic drugs or other drugs used in this experiment.

### 2.3 Randomization and Blinding

Eligible participants were randomly assigned (1:1) to the active rTMS or sham rTMS group using block randomization (block size = 6). The randomization sequence was generated using a computer‐based program by an independent researcher of the team who was not involved in participant recruitment, intervention delivery, outcome assessment, or data analysis. Allocation concealment was ensured using sequentially numbered, opaque, sealed envelopes. After enrollment and baseline assessment, the envelope was opened at the time of the first rTMS session to determine group assignment.

This trial was designed to be double‐blind. Participants, anesthesiologists, surgeons, nurses, and outcome assessors were blinded to treatment allocation. Sham stimulation was delivered using a tilted‐coil method to mimic the setup and acoustic click of active stimulation while minimizing cortical stimulation. The rTMS operator did not participate in outcome assessment, data collection, or statistical analysis. Sham stimulation was delivered using a tilted‐coil approach; therefore, the rTMS operator was not blinded to group allocation. However, the operator had no role in outcome assessment or statistical analysis.

### 2.4 rTMS Procedure

**FIGURE 1:**
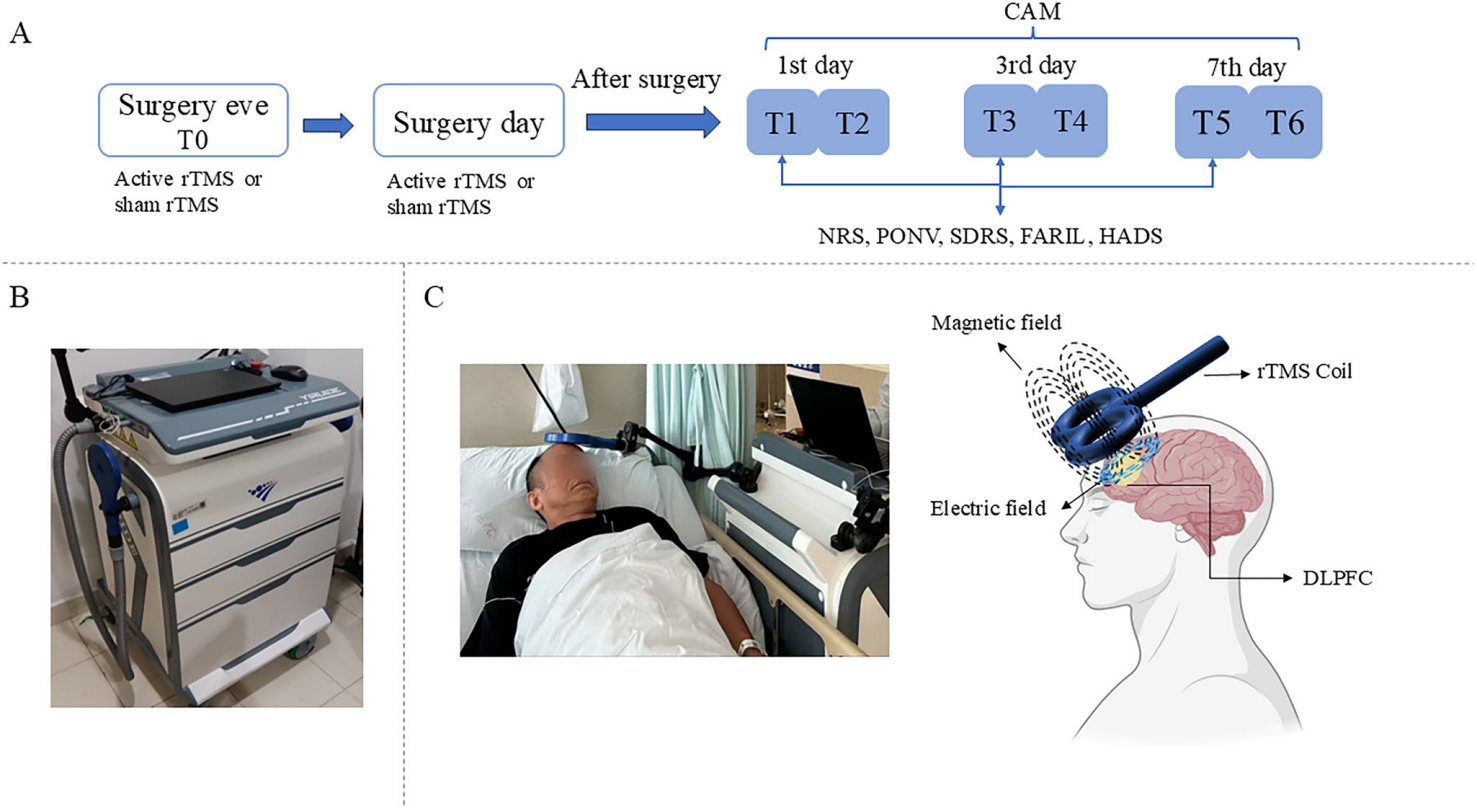
**The timeline of the trial and apparatus used in this test**. (A) The experimental design and timeline of the two experimental sessions (active rTMS and sham rTMS). (B) The apparatus used in this trial. (C) The rTMS coil location (Partially created in BioRender.com). rTMS, repetitive transcranial magnetic stimulation; CAM, Confusion Assessment Method; NRS, Numeric Rating Scale score; PONV, postoperative nausea and vomiting; SDRS, Sleep Dysfunction Rating Scale; HADS, Hospital Anxiety and Depression Scale.

The rTMS intervention uses the figure‐of‐eight coil of the Transcranial Magnetic Stimulator (machine manufacturer: Wuhan Yiruide Medical Equipment New Technology Co., Ltd; machine specification model: Mag TD), as shown in Figure 1B, C. The study targeted the left DLPFC for stimulation (Kumar et al. 2017; Pagali et al. 2024), using a cap designed based on the EEG 10–20 system for positioning. The DLPFC was located 5 cm anterior to the parasagittal area corresponding to the resting motor threshold (RMT). The coil was placed over the left DLPFC position, tangent to the scalp. Before treatment, the RMT of the right abductor pollicis brevis muscle was measured using motor evoked potentials (MEPs) (Rossini et al. 2015). RMT is defined as the minimum intensity required to evoke MEPs greater than 50 µV in ≥ 5 of 10 trials while the muscle is in a relaxed state (Rossini et al. 2015). In this study, the observational method was used, where the minimum stimulation intensity was set at the threshold that elicited at least 5 right thumb movements out of 10 stimuli. The actual stimulus intensity was calculated as 110% of the individual's RMT. rTMS treatment was administered at a frequency of 10 Hz and 110% of RMT (Li et al. 2023; Sabbagh et al. 2020). Each stimulation lasted 2 s, with an inter‐stimulation interval of 20 s. A total of 1080 pulses were delivered over a treatment duration of 20 min. Participants received two sessions of rTMS therapy: one on the day prior to surgery and another on the day of surgery, with a minimum interval of 12 h between the sessions. Patients in the sham rTMS group underwent the same treatment regimen with identical parameters. The coil was held at approximately 90° to the scalp (tilted‐coil sham) to mimic the acoustic click while minimizing effective cortical stimulation. The researchers closely monitored patients for any signs of discomfort during the treatment. If the patient experienced unbearable discomfort or serious adverse events, the stimulation was immediately stopped, and the event was documented. The rTMS treatment process adhered to established safety guidelines (Lefaucheur et al. 2020; Rossi et al. 2021).

### 2.5 Anesthesia Procedures

After the patient entered the operating room, standard monitoring procedures were performed, including electrocardiography, non‐invasive blood pressure, pulse oximetry, and bispectral index (BIS) monitoring. Following local infiltration anesthesia with lidocaine, radial artery catheterization was performed to monitor blood pressure in the invasive artery. General anesthesia was administered, with induction using propofol (1.5–2.0 mg/kg), sufentanil (0.15–0.5 µg/kg), and rocuronium (0.6–0.8 mg/kg). Endotracheal intubation was performed after the BIS value dropped below 60, followed by mechanical ventilation using the anesthesia machine. During surgery, remifentanil was administered as an intravenous infusion at a rate of 0.1–2.0 µg/kg/min, combined with continuous sevoflurane inhalation to maintain an alveolar concentration of 1.0–1.5 and a BIS value between 40 and 60 by adjusting the anesthetic dose. Vasoactive drugs were administered as needed to maintain hemodynamic stability during anesthesia. After surgery, the patient was transferred to the Post Anesthesia Care Unit (PACU) or the Anesthesia Intensive Care Unit (AICU). Once the patient regained consciousness, optimal tidal volume was ensured, and the endotracheal tube was removed. In addition, a single intravenous bolus of sufentanil (5 µg) was administered within 30 min before the end of surgery, and the patient‐controlled intravenous analgesia (PCIA) pump was connected immediately after surgery. Postoperative analgesia was managed with PCIA pump containing 1.5–2.0 µg/kg sufentanil and 8 mg ondansetron in 100 mL of normal saline, with a background infusion rate of 2.0 mL/h, a self‐administered dose of 1.0 mL and the occlusion interval was 15 min.

### 2.6 Clinical Outcomes and Assessments

The primary outcome measure was the incidence of POD within 7 postoperative days. POD was assessed using the Confusion Assessment Method (CAM) for non‐intubated patients and the Confusion Assessment Method for Intensive Care Unit (CAM‐ICU) for intubated patients (Ely et al. 2001; Inouye et al. 1990). Assessments were conducted by professional researchers who were blinded to group assignments. The CAM has high sensitivity (94%‐100%) and specificity (90%‐95%) and is suitable for use by non‐psychiatrists in delirium evaluation. The assessment focused on four aspects: acute changes or fluctuations in consciousness, inattention, disorganized thinking, and altered level of consciousness. POD was assessed twice a day on the postoperative days 1, 3, and 7, with at least a 6‐h interval between assessments. The assessment times were as follows: the morning of the first day (T1), the afternoon of the first day (T2), the morning of the third day (T3), the afternoon of the third day (T4), the morning of the seventh day (T5), and the afternoon of the seventh day (T6). If delirium was identified, the delirium assessment was performed daily until the symptoms disappeared.

Secondary outcome measures included the delirium subtype (Robinson et al. 2011) (hyperactivity delirium characterized by agitation, restlessness, hallucinations, and aggression; hypoactive delirium characterized by drowsiness, difficulty concentrating, and psychomotor retardation; mixed delirium, defined as fluctuations between hypoactive and hyperactive presentations); delirium severity (assessed by Delirium Rating Scale Revised‐98 [DRS‐R‐98]) (de Rooij et al. 2006); pain intensity (assessed by Numeric Rating Scale [NRS] at T1, T3 and T5); sleep quality (assessed by Sleep Dysfunction Rating Scale [SDRS] at T1, T3, and T5); postoperative nausea and vomiting (PONV, assessed by NRS at T1, T3, and T5); frailty (assessed by FRAIL scale at T1, T3, and T5); anxiety and depression (assessed by Hospital Anxiety and Depression Scale [HADS] at T3 and T5). Due to the fluctuating nature of delirium, the researchers conducting the assessments also asked the patient's family and caregivers about the patient's symptoms and reviewed medical records.

The timeline of this trial is shown in Figure 1A.

### 2.7 Sample Size

The sample size was determined a priori using PASS 15.0 (NCSS, LLC, Kaysville, UT, USA). The incidence of POD within 7 days after surgery was the primary outcome measure. Referring to the results of previous studies, the incidence of POD in elderly surgery patients was 9.0%–45.0% (Daiello et al. 2019; Gleason et al. 2015). We assumed that the incidence of POD could be reduced from 25% in the control group to 11.1% in the intervention group. With a study power of 0.80 and a significance level of 0.05 (two‐sided), we calculated that 115 patients per group. Considering a 10% loss to follow‐up, the sample size was increased to 127 per group.

### 2.8 Statistical Analysis

Since the overall proportion of missing data was below 5%, no imputation of missing data was performed. All analyses were conducted with the intention‐to‐treat (ITT) principle. Per‐protocol (PP) analyses of the primary outcome were additionally conducted as sensitivity analyses to assess the robustness of the findings.

The Kolmogorov–Smirnov test was used to assess normality. The independent sample *t*‐test was used for normally distributed data, and the Mann–Whitney *U* test was used otherwise for nonnormally distributed data. Continuous variables with normal distribution were represented by mean (SD), and variables with nonnormal distribution were represented by median (IQR). Categorical variables were expressed as frequencies and proportions and analyzed using the χ^2^ test or Fisher exact test. The primary outcome, the incidence of POD within 7 days after surgery was compared using the χ^2^ test or Fisher exact test, with differences between groups expressed as relative risk (RR) and 95% confidence intervals (CIs). The cumulative incidence of POD was analyzed with Kaplan–Meier survival analyses and the between‐group difference of incidence was compared with the Log‐rank test. Data collected at multiple points in time were analyzed using repeated‐measures analysis of variance (ANOVA). Non‐normally distributed continuous data were analyzed with nonparametric tests. Intra‐group temporal changes were evaluated by the Friedman test followed by Bonferroni‐adjusted Wilcoxon tests. Inter‐group comparisons at each time point were made using the Mann–Whitney *U* test with Bonferroni correction. Univariable and multivariable logistic regression models were employed to assess the associations between variables and postoperative delirium. Variables with a univariable *p* value < 0.1 or clinical importance were candidates for the multivariable model. Multicollinearity was assessed using variance inflation factors (VIF). Adjusted odds ratios (aORs) with 95% CIs were calculated using multivariable logistic regression.

In the exploratory analysis, a post hoc subgroup analysis was conducted. Within each subgroup, adjusted aORs were calculated using the core multivariable model. Effect heterogeneity was formally tested by evaluating the significance of interaction terms. A mediation analysis was further performed. Model 4 of the PROCESS macro was used for the mediation analysis and controlled covariates. The indirect effect was estimated using bootstrapping with a 95% CI. If the CI includes zero, the mediation effect is not significant at the 5% level.

All analyses were performed using IBM SPSS Statistics version 25.0 (IBM Corporation, USA). Statistical significance was set at a two‐sided *p* < 0.05.

## 3 Results

### 3.1 Baseline Demographics and Clinical Characteristics

**FIGURE 2:**
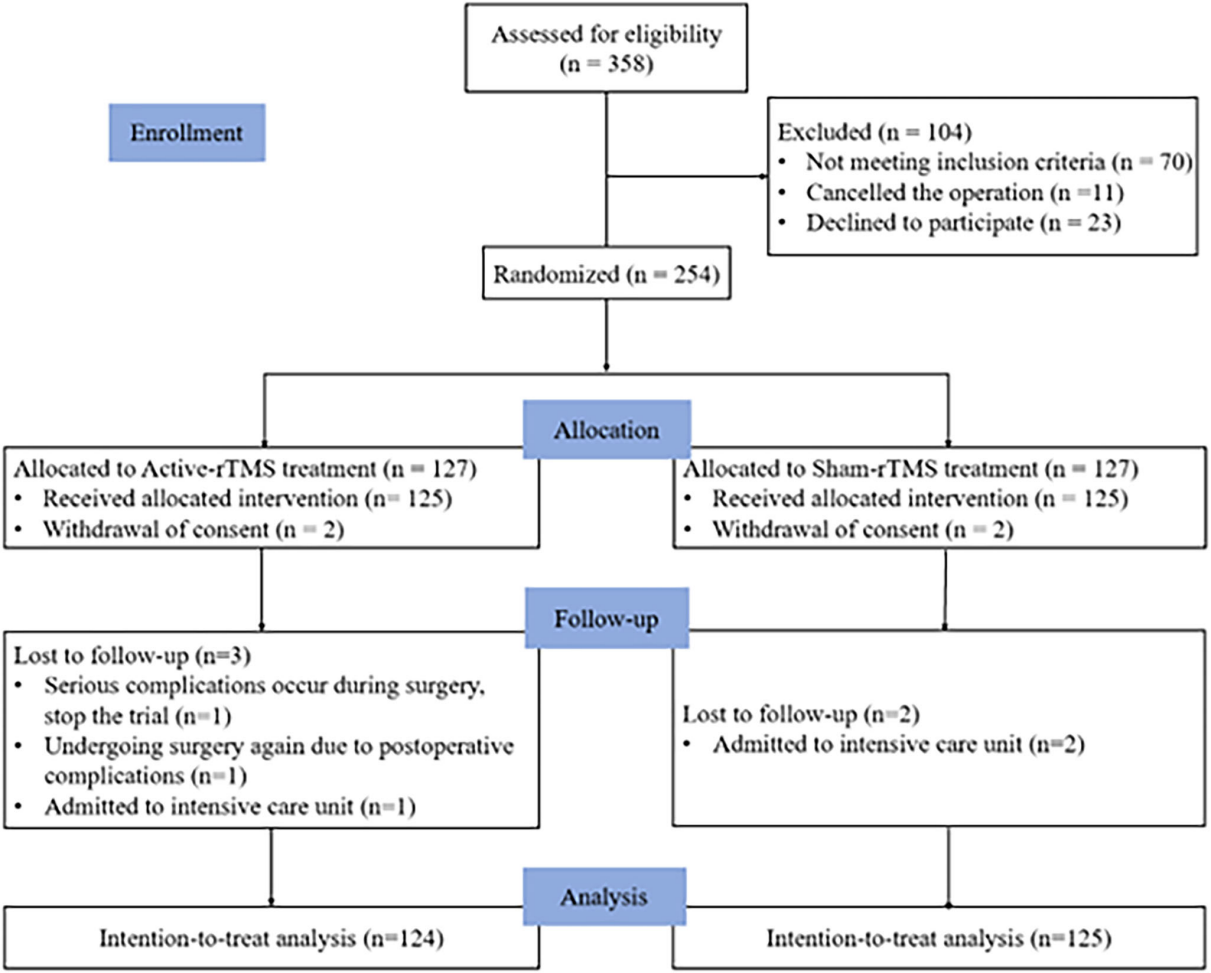
CONSORT flow diagram.

The participant flow chart is shown in Figure 2. A total of 358 patients were screened, with 104 excluded: 70 did not meet the inclusion criteria (6 had an MMSE score below 17, 25 had metal implants in their bodies, 4 had Parkinson's disease, 3 had Alzheimer's disease, 5 had psychiatric disorders, 27 had neurological disorders), 11 canceled surgeries, and 23 declined to participate. Finally, 254 patients were enrolled and randomly assigned to either active‐rTMS group (*n* = 127) or sham‐rTMS group (*n* = 127). Notably, 2 patients in each of the two groups refused to receive the second preoperative intervention. In the active‐rTMS group, 1 patient had serious intraoperative complications and the experiment was stopped. In addition, 1 patient in the active‐rTMS group had serious complications after surgery and was transferred to the ICU, 1 patient was transferred to the ICU after surgery, and 2 patients in the control group were transferred to the ICU and could not be evaluated in time, so they were classified as lost to follow‐up. To enhance study reliability and validity, an ITTanalysis was conducted on all 249 randomized patients, with demographic and clinical data shown in Table 1. A PP analysis was performed on the 245 patients who completed the study, with corresponding data presented in eTable S1. Baseline and clinical characteristics were well balanced between groups in both the ITT (Table 1 and eTable S1) and PP analyses (Table 2 and eTable S2). Intraoperative and postoperative data were also comparable between the groups.

**TABLE 1:** Baseline characteristics of study participants.

Characteristic	Active‐rTMS (*n* = 124)	Sham‐rTMS (*n* = 125)	*p* value
Age, median (IQR), year	69.5 (63.3–72.8)	68.0 (63.0–73.0)	0.829
Sex, No. (%)			0.660
Male	65 (52.4)	69 (55.2)	
Female	59 (47.6)	56 (44.8)	
BMI, mean (SD), kg/m^2^	23.4 (3.1)	23.9 (3.5)	0.240
Education level, No. (%)			0.523
Illiteracy	41 (33.1)	32 (25.6)	
Elementary school	39 (31.5)	51 (40.8)	
Middle school	31 (25.0)	28 (23.7)	
High school	9 (7.3)	11 (8.8)	
College graduate	4 (3.2)	3 (2.4)	
ASA classification, No. (%)			0.250
II	83 (66.9)	92 (73.6)	
III	41 (33.1)	33 (26.4)	
Type of operation, No. (%)			0.145
Abdominal surgery	59 (47.6)	75 (60.0)	
Orthopedic surgery	43 (34.7)	33 (26.4)	
Thoracic surgery	22 (17.7)	17 (13.6)	
MMSE score, median (IQR)	23.0 (22.25–25.75)	22.0 (20.0–25.0)	0.476
SDRS score, median (IQR)	5.0 (5.0–7.0)	6.0 (5.0–7.5)	0.295
HADS‐A score, median (IQR)	2.0 (0.0–2.0)	2.0 (0.0–2.0)	0.368
HADS‐D score, median (IQR)	0.0 (0.0–1.0)	0.0 (0.0–1.0)	0.636
FRAIL scores, median (IQR)	0.0 (0.0–0.0)	0.0 (0.0–0.0)	0.188
Smoking, No. (%)	32 (25.8)	27 (21.6)	0.435
Drinking, No. (%)	18 (14.5)	17 (13.6)	0.835
Comorbidities, No. (%)			0.391
Hypertension	61 (49.2)	69 (53.1)	
Diabetes	21 (47.7)	23 (52.3)	
Cardiovascular diseases	9 (7.3)	11 (8.8)	
Respiratory diseases	8 (6.5)	3 (2.4)	
History of malignancy	14 (11.3)	22 (17.6)	
TG, median (IQR), mmol/L	1.5 (1.1–2.2)	1.5 (1.1–2.2)	0.635
TC, mean (SD), mmol/L	4.7 (1.1)	4.5 (0.9)	0.270
LDL‐C, mean (SD), mmol/L	2.7 (0.8)	2.6 (0.7)	0.101
HDL‐C, median (IQR), mmol/L	1.3 (1.1–1.5)	1.3 (1.1–1.5)	0.812

**Abbreviations**: ASA, American Society of Anesthesiologists physical status classification; BMI, body mass index (calculated as weight in kilograms divided by height in meters squared); FRAIL, FRAIL Scale; HADS‐A, Hospital Anxiety and Depression Scale—Anxiety; HADS‐D, Hospital Anxiety and Depression Scale—Depression; IQR, interquartile range; MMSE, Mini‐Mental State Examination score; rTMS, repetitive transcranial magnetic stimulation; SD, standard deviation; SDRS, Sleep Dysfunction Rating Scale; TC, Total cholesterol; TG, triglyceride.

**TABLE 2:** Intraoperative and postoperative data among two groups.

Characteristic	Active‐rTMS (*n* = 124)	Sham‐rTMS (*n* = 125)	*p* value
**Intraoperative**			
Duration of surgery, median (IQR), min	155.0 (125.0–196.3)	153.0 (125.5–202.5)	0.772
Duration of anesthesia, median (IQR), min	175.0 (150.0–222.3)	174.0 (144.5–228.0)	0.756
Extubation time, median (IQR), min	42.5 (25.0–94.5)	41.0 (26.0–96.0)	0.814
Infusion quantity, median (IQR), mL	1500 (1000.0–2000.0)	1500 (1000.0–2000.0)	0.136
Estimated blood loss, median (IQR), mL	50.0 (20.0–100.0)	50.0 (20.0–100.0)	0.529
**Postoperative**			
In‐hospital delirium, No. (%)	10 (8.1)	36 (28.8)	<0.001
Worst delirium severity, mean (SD)	21.3 (1.2)	20.9 (2.1)	0.270
Type of delirium, No. (%)			0.437
Hypoactive	0 (0.0)	3 (2.4)	
Hyperactive	8 (6.5)	28 (22.4)	
Mixed	2 (1.6)	5 (4.0)	
NRS score for pain, median (IQR)			
T1	2 (2.0–3.0)	3 (2.0–3.0)	<0.001
T3	2 (1.0–2.0)	2 (2.0–2.0)	<0.001
T5	0 (0.0–1.0)	0 (0.0–1.0)	0.023
SDRS score, median (IQR)			
T1	10 (9.0–11.0)	11 (10.0–13.0)	<0.001
T3	8 (7.0–9.0)	9 (8.0–11.0)	<0.001
T5	6 (5.0–7.0)	7 (6.0–8.0)	0.017
NRS score for PONV, median (IQR)			
T1	0 (0.0–1.0)	0 (0.0–2.0)	0.321
T3	0 (0.0–0.0)	0 (0.0–0.0)	0.208
T5	0 (0.0–0.0)	0 (0.0–0.0)	>0.999
FRAIL scores, median (IQR)			
T1	2 (2.0–3.0)	3 (2.0–3.0)	0.001
T3	2 (1.0–2.0)	2 (2.0–2.0)	0.037
T5	1 (0.0–1.0)	1 (1.0–2.0)	<0.001
HADS‐A score, median (IQR)			
T3	2 (2.0–3.0)	3 (2.0–3.0)	<0.001
T5	1 (0.0–2.0)	2 (1.0–2.0)	<0.001
HADS‐D score, median (IQR)			
T3	2 (1.0–2.0)	2 (1.0–3.0)	<0.001
T5	0 (0.0–0.0)	0 (0.0–1.0)	<0.001
CRP, median (IQR), mg/L	32.8 (15.5–53.7)	37.2 (21.7–59.6)	0.122
Duration of hospitalization, median (IQR), day			
Total	12.0 (8.0–17.0)	12.0 (9.0–16.5)	0.800
After surgery	8.0 (6.0–12.0)	8.0 (7.0–11.0)	0.730

*Note: p* values are from two‐sided Mann–Whitney *U* tests for between‐group comparisons at each time point. For comparisons across three postoperative time points, statistical significance was interpreted using a Bonferroni‐adjusted threshold of *p* < 0.017 (0.05/3). Categorized score distributions for pain and PONV are provided in eTable S3.

**Abbreviations**: HADS‐A, Hospital Anxiety and Depression Scale—Anxiety; HADS‐D, Hospital Anxiety and Depression Scale—Depression; IQR, interquartile range; NRS, Numeric Rating Scale score; PONV, postoperative nausea and vomiting; rTMS, repetitive transcranial magnetic stimulation; SD, standard deviation; SDRS, Sleep Dysfunction Rating Scale.

### 3.2 Primary Outcome

**FIGURE 3:**
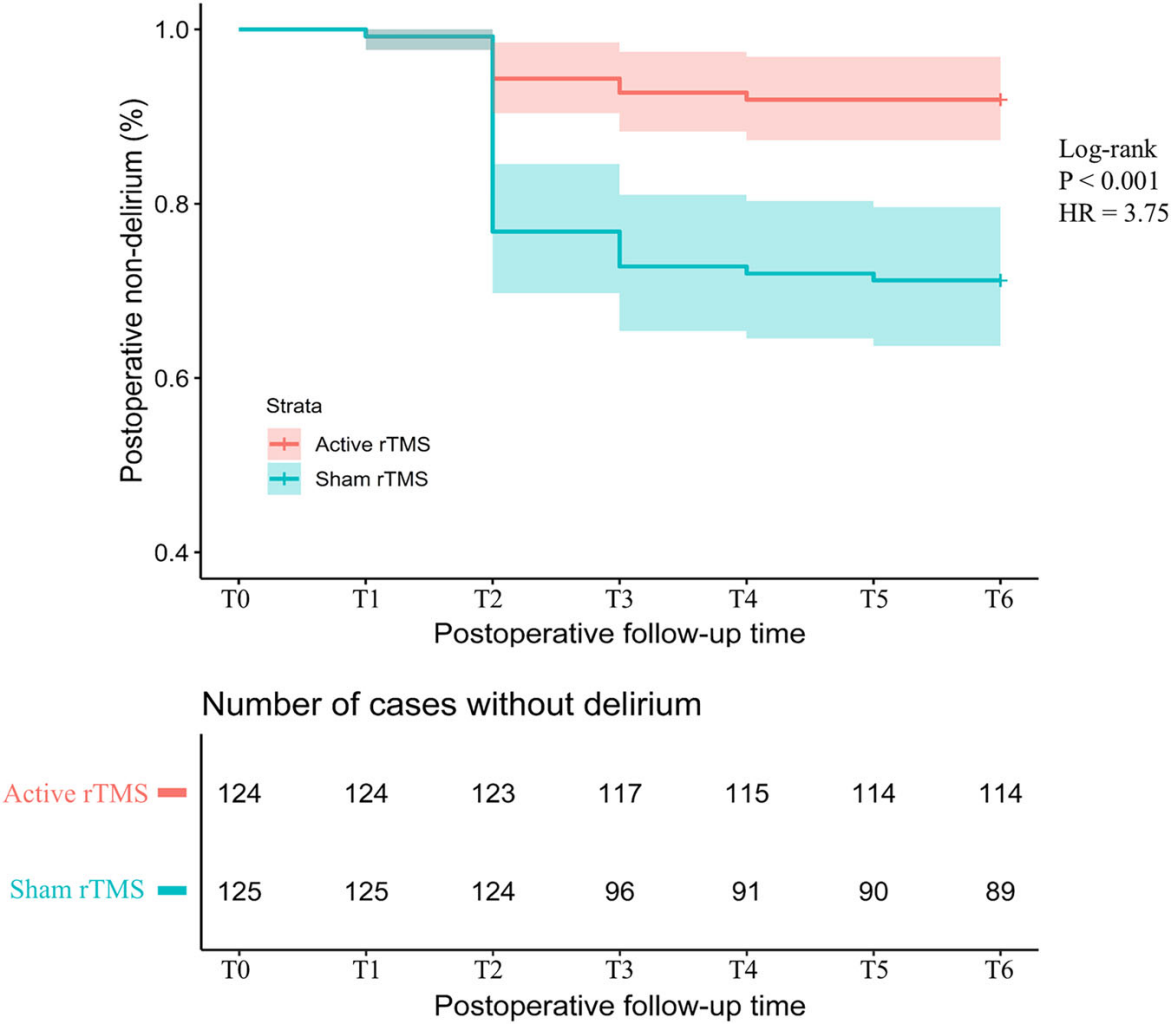
**Kaplan–Meier curve showing intention‐to‐treat analysis of the cumulative incidence of postoperative non‐delirium during postoperative days 1 to 7 in the two groups**. Abbreviations: rTMS, repetitive transcranial magnetic stimulation; HR, hazard ratio.

All included patients followed a standardized procedure according to the trial protocol and as shown in Figure 1A. In the ITT population (*n* = 249), the incidence of POD at any time within 7 days after surgery was significantly lower in the active rTMS group (10 of 124 [8.1%]) than that in the sham rTMS group (36 of 125 [28.8%]; RR, 0.22; 95% CI 0.10 to 0.46; *p* < 0.001; Table 2 and Figure 3). The overall incidence of POD was 18.5% (46 of 249 patients).

In the PP population (*n* = 245), the results were consistent with the ITT analysis. The incidence of POD was significantly lower in the active rTMS group (10 of 122 [8.2%]) than that in the sham rTMS group (35 of 123 [28.5%]; RR, 0.29; 95% CI 0.15 to 0.56; *p* < 0.001; eTable S2). The overall incidence of POD was 18.4% (45 of 245 patients).

### 3.3 Secondary Outcomes

Table 2 gives the clinical postoperative outcomes. The ITT analysis showed no statistically significant difference in the worst delirium severity score (overall: mean, 21.1 [SD, 1.7]; *p* = 0.270) of delirium patients between the sham rTMS and active groups. In the active rTMS group, the incidence of all three delirium subtypes was lower, but the composition ratios showed no significant difference.

Other secondary outcomes, including pain, sleep disturbance (SDRS), depression (HADS‐D), anxiety (HADS‐A), and frailty (FRAIL) scores, improved significantly over time in both groups (all *p* < 0.001), with all post hoc pairwise comparisons remaining significant after Bonferroni correction.

Between‐group comparisons showed that the active rTMS group had significantly lower scores than the sham group in pain scores at T1 (2 [IQR, 2.0 to 3.0] vs. 3 [IQR, 2.0 to 3.0], *p* < 0.001) and T3 (2 [IQR, 1.0 to 2.0] vs. 2 [IQR, 2.0 to 2.0], *p* < 0.001), SDRS scores at T1 (10 [IQR, 9.0 to 11.0] vs. 11 [IQR, 10.0 to 13.0], *p* < 0.001) and T3 (8 [IQR, 7.0 to 9.0] vs. 9 [IQR, 8.0 to 11.0], *p* < 0.001), anxiety and depression scores at T3 (HADS‐A: 2 [IQR, 2.0 to 3.0] vs. 3 [IQR, 2.0 to 3.0], *p* < 0.001; HADS‐D: 2 [IQR, 1.0 to 2.0] vs. 2 [IQR, 1.0 to 3.0], *p* < 0.001) and T5 (HADS‐A: 1 [IQR, 0.0 to 2.0] vs. 2 [IQR, 1.0 to 2.0], *p* < 0.001; HADS‐D: 0 [IQR, 0.0 to 0.0] vs. 0 [IQR, 0.0 to 1.0], *p* < 0.001), and FRAIL scores at T1 (2 [IQR, 2.0 to 3.0] vs. 3 [IQR, 2.0 to 3.0], *p* < 0.001) and T5 (1 [IQR, 0.0 to 1.0] vs. 1 [IQR, 1.0 to 2.0], *p* < 0.001). However, between‐group differences were not sustained at later assessments for pain at T5 (0 [IQR, 0.0 to 1.0] vs. 0 [IQR, 0.0 to 1.0], *p* = 0.023) and SDRS at T5 (6 [IQR, 5.0 to 7.0] vs. 7 [IQR, 6.0 to 8.0], *p* = 0.017), and the T3 (2 [IQR, 1.0 to 2.0] vs. 2 [IQR, 2.0 to 2.0], *p* = 0.037) difference in frailty was not significant after correction. Additionally, no significant between‐group differences were observed in PONV scores at any time point (T1: 0 [IQR, 0.0 to 1.0] vs. 0 [IQR, 0.0 to 2.0], *p* = 0.321; T3: 0 [IQR, 0.0 to 0.0] vs. 0 [IQR, 0.0 to 0.0], *p* = 0.208; T7: 0 [IQR, 0.0 to 0.0] vs. 0 [IQR, 0.0 to 0.0], *p* = 0.158; Mann–Whitney *U* tests with Bonferroni correction). Compared with the sham rTMS group, there was no significant difference between the two groups, although the active rTMS group had lower CRP level on the first day after surgery (32.8 [IQR, 15.5 to 53.7] vs. 37.2 [IQR, 21.7 to 59.6], *p* = 0.122).

Single factor logistic regression analysis showed that the *p*‐values of rTMS, sex, age, ASA classification, education level, MMSE scores, duration of anesthesia, and extubation time are less than 0.1 (eTable S4). Incorporating these factors into the multivariate logistic regression analysis, we found that active rTMS intervention showed significant statistical differences (aOR, 0.15; 95% CI 0.06 to 0.36; *p* < 0.001) (eTable S4). Additionally, Multivariable analysis identified higher MMSE score (aOR 0.57, 95% CI 0.44 to 0.73; *p* < 0.001) also as independent protective factors. Multicollinearity was assessed by variance inflation factors (VIF), and all variables included in the multivariate model had a VIF < 5, indicating no severe multicollinearity.

To explore the consistency of the rTMS treatment effect, a post hoc subgroup analysis was performed. As shown in eFigure S1, the active rTMS group consistently demonstrated a trend toward a reduced risk of postoperative delirium across all predefined subgroups, with effect estimates favoring the intervention in line with the primary analysis. No significant interaction was observed between the treatment effect and age, ASA classification, education level, type of surgery, or baseline MMSE score (all *p* for interaction > 0.05).

### 3.4 Perioperative Conditions

There were no significant differences in the duration of operation (155.0 [IQR, 125.0 to 196.3] min vs. 153.0 [IQR, 125.5 to 202.5] min; *p* = 0.772), the duration of anesthesia (175.0 [IQR, 150.0 to 222.3] min vs. 174.0 [IQR, 144.5 to 228.0] min; *p* = 0.756), extubation time (42.5 [IQR, 25.0 to 94.5] min vs. 41.0 [IQR, 26.0 to 96.0] min; *p* = 0.814), infusion quantity (1500.0 [IQR, 1000.0 to 2000.0] mL vs. 1500.0 [IQR, 1000.0 to 2000.0] mL; *p* = 0.136), estimated blood loss (50.0 [IQR, 20.0 to 100.0] mL vs. 50.0 [IQR, 20.0 to 100.0] mL; *p* = 0.529), and duration of hospitalization (12.0 [IQR, 8.0 to 17.0] days vs. 12.0 [IQR, 9.0 to 16.5] days; *p* = 0.800) between the active rTMS and sham rTMS group (Table 2).

### 3.5 Mediation Effect Analysis

**FIGURE 4:**
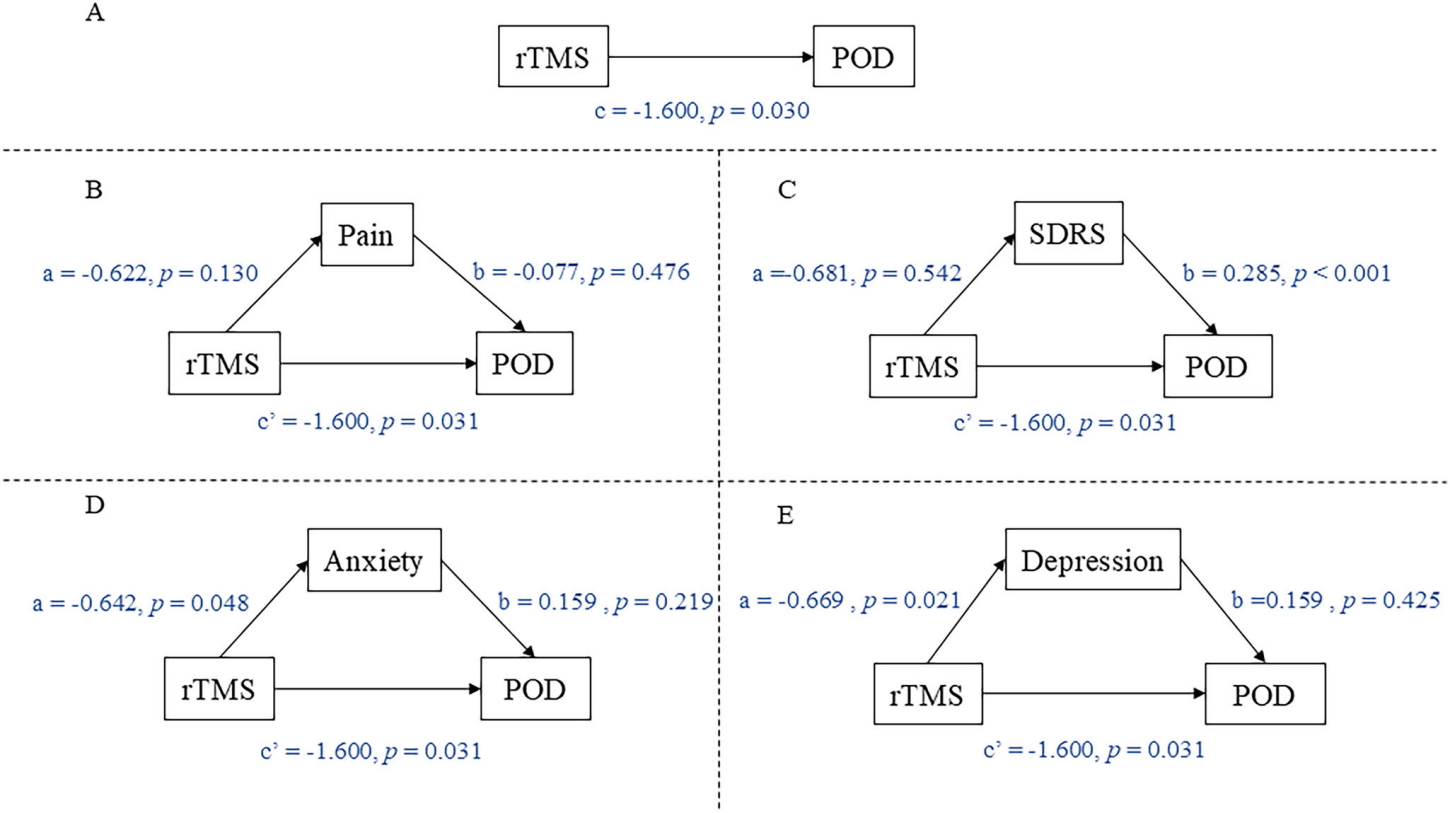
**Proposed models that investigate mediated effects. Abbreviations**: rTMS, repetitive transcranial magnetic stimulation; POD, postoperative delirium; SDRS, Sleep Dysfunction Rating Scale; Pain, Numeric Rating Scale score for pain; SDRS, Sleep Dysfunction Rating Scale; Anxiety, Hospital Anxiety and Depression Scale to Anxiety score; Depression, Hospital Anxiety and Depression Scale to Depression score.

To further investigate whether rTMS indirectly affects POD by relieving pain, sleep disorders, anxiety, or depression, we performed a mediation analysis of the data. First, the area under the curve (AUC) of each patient's pain scores, SDRS scores, anxiety scores, and depression scores were calculated, and then brought into the model as mediating variables separately. The mediation analysis was performed using one independent variable (rTMS), one dependent variable (POD), and one mediator. Each of the above four values was treated as a mediator value and brought into the mediational model for calculation. There was no significant difference in the mediating effect of all four variables, including pain, anxiety, depression, and sleep disorders (eTable S5; Figure 4B‐E).

### 3.6 Adverse Events and Safety

All patients tolerated rTMS treatment well, with no serious adverse reactions, and there was no statistical difference in the incidence of adverse events between the two groups (24 of 124 [19.4%] vs. 14 of 125 [11.2%], *p* = 0.074; Table 3). Common adverse reactions are shown in the table, including mild headache (5 in the active rTMS group and 2 in the sham rTMS group), scalp discomfort (11 in the active rTMS group and 7 in the sham rTMS group), muscle twitching (5 in the active rTMS group and 4 in the sham rTMS group), dizziness (1 in the active rTMS group), and neck discomfort (1 in the active rTMS group).

**TABLE 3:** Adverse events reported.

Adverse events	Active rTMS (*n* = 124)	Sham rTMS (*n* = 125)	*p* value
Total, No. (%)	23 (19.4)	13 (11.2)	0.074
Mild headache	5 (4.0)	2 (1.6)	
Scalp discomfort	11 (8.9)	7 (5.6)	
Muscle twitching	5 (4.0)	4 (3.2)	
Dizziness	2 (1.6)	1 (0.8)	
Neck discomfort	1 (0.8)	0 (0.0)	

## 4 Discussion

In this single‐center, prospective randomized controlled trial, we found that two sessions of rTMS applied to the left DLPFC before surgery significantly reduced the incidence of POD in elderly patients undergoing non‐cardiac surgery. In addition, the study indicated that active rTMS can also alleviate pain in the early postoperative period, decrease anxiety and depression scores, and reduce the occurrence of frailty in elderly patients. This trial also suggested that patients with lower preoperative MMSE scores (MMSE < 27) may have an increased risk of developing POD.

In recent years, numerous studies have demonstrated that rTMS has beneficial effects on cognitive impairment (Pagali et al. 2024), including MCI, AD, and post‐stroke cognitive dysfunction (PSCI). However, the application of rTMS in perioperative medicine has been less extensively explored. To date, only one randomized study has demonstrated that early postoperative rTMS can reduce the incidence of POD in patients with major abdominal surgery (Zhou et al. 2025). Extending this literature, our trial indicated that preoperative rTMS intervention exerts preventive effects against POD in elderly patients undergoing elective non‐cardiac surgery.

Based on previous studies (Jung et al. 2022; Li et al. 2023; Pagali et al. 2024), we selected the left DLPFC as the stimulation target and 10 Hz as the stimulation frequency. Zhou et al. (2025) reported that 10 Hz rTMS targeting the left DLPFC reduced the incidence of POD in elderly patients undergoing abdominal surgery. In line with this finding, our results indicated that 10 Hz rTMS targeting the left DLPFC similarly reduced POD incidence in elderly patients undergoing non‐cardiac surgery. However, given the limited number of studies, no consensus has yet been reached regarding the optimal rTMS parameters for perioperative reduction of POD incidence.

The pathogenesis of POD remains incompletely understood, and the mechanisms by which rTMS may reduce POD are likely multifactorial. Conceptually, preoperative HF‐rTMS targeting the left DLPFC may lower POD risk by (i) enhancing synaptic plasticity and modulating prefrontal control networks, (ii) attenuating perioperative neuroinflammation, and (iii) stabilizing neurotransmitter systems involved in arousal and cognition. We discuss these potential pathways below.

Neuroinflammation is widely implicated in POD. Inflammaging in older adults promotes chronic low‐grade inflammation, which may impair neuroplasticity and increase vulnerability to perioperative brain injury (Bugiani 2021). Perioperative stress amplifies systemic inflammation, disrupts the BBB, and worsens neuronal injury (Alam et al. 2018; Brattinga et al. 2024), and BBB dysfunction has been linked to delirium (Devinney et al. 2023). Preclinical and early clinical evidence indicates that HF‐rTMS over the DLPFC may reduce pro‐inflammatory cytokines and modulate glial activity, thereby mitigating neuroinflammation and supporting synaptic plasticity and cognition (Antonioni et al. 2025; Cha et al. 2022; Zuo et al. 2022). This anti‐inflammatory action provides a plausible mechanism for the protective effect of rTMS on POD.

Another mechanism involves cortical plasticity and network‐level modulation. Cortical plasticity underlies learning and memory (Mansvelder et al. 2019), and the DLPFC plays a central role in working memory and executive control through its connectivity with large‐scale brain networks (Kumar et al. 2017). HF‐rTMS may induce LTP‐like plasticity within prefrontal circuits and strengthen frontoparietal control circuitry, which may improve regulation of the default mode network that is closely related to attention and executive control (Corlier et al. 2020; Liston et al. 2014). Additionally, HF‐rTMS may increase cerebral perfusion and metabolism, and upregulate neurotrophic signaling, changes that support reorganization of cognition‐related circuits (Han et al. 2023; Kumar et al. 2021; Wu et al. 2021).

In addition, neurotransmitter imbalance is another established contributor to delirium. Perioperative drug exposure and surgical stress may disrupt neurotransmitter systems, leading to acute disturbances in cognition, attention, and arousal and thereby increasing POD risk (Wilson et al. 2020). Cholinergic deficiency, as well as dysregulation of histaminergic and noradrenergic systems, has been linked to impaired arousal regulation and prefrontal dysfunction across delirium phenotypes (Arnsten 2009; Aston‐Jones and Cohen 2005; Scammell et al. 2019; Wilson et al. 2020). rTMS may modulate multiple neurotransmitter pathways by influencing transmitter release and synaptic efficacy, which could help stabilize cognition‐arousal networks in the perioperative period (Antonioni et al. 2025). Taken together, POD is unlikely to be driven by a single mechanism; rather, it arises from the interaction of multiple perioperative stressors and neurobiological pathways, and rTMS may act on several of these pathways simultaneously.

In addition to reducing the incidence of POD, rTMS appeared to influence other perioperative outcomes, including pain, anxiety, depression, sleep quality, and frailty. Postoperative pain and increased opioid use have been associated with a higher risk of delirium (Aldecoa et al. 2024; Leung et al. 2013), whereas adequate analgesia may reduce POD incidence (Peden et al. 2021). In this trial, active rTMS targeting the left DLPFC was associated with significantly lower early postoperative pain scores, whereas the between‐group separation was smaller by postoperative day 7 (Table 2). Although most studies indicate that the motor cortex (M1) is the primary stimulation site for analgesia (Lefaucheur et al. 2020; Zhou et al. 2024), DLPFC stimulation has also been linked to short‐term pain relief (Che et al. 2021). A plausible explanation is that prefrontal rTMS may transiently influence prefrontal‐limbic and descending pain‐modulatory circuits and related functional connectivity (Li et al. 2024). However, sustained analgesic effects may require more sessions, and standardized postoperative analgesic management may also reduce between‐group differences as recovery progresses.

Regarding postoperative pain, rTMS was also associated with lower sleep disturbance scores in the early postoperative period (Table 2), consistent with previous evidence of its beneficial effects on sleep quality (Lanza et al. 2023; Liu et al. 2025). The association between sleep disturbances and PND has received growing attention (Fadayomi et al. 2018; O'Gara et al. 2021), possibly through elevated perioperative inflammatory activity (Fadayomi et al. 2018). Therefore, short‐term improvements in sleep may represent an additional pathway contributing to delirium prevention. Anxiety and depression are common perioperative stress responses that may exacerbate inflammatory activity and disrupt sleep, thereby increasing vulnerability to POD (Yang et al. 2023). In this trial, HF‐rTMS was associated with significantly lower postoperative anxiety and depression scores (Table 2), in line with prior evidence supporting its efficacy in mood regulation (Fu et al. 2025; George et al. 2023; Kan et al. 2023). Interestingly, improvements in anxiety and depression scores remained detectable through postoperative day 7, which may indicate a relatively more sustained effect of prefrontal network modulation on affective regulation. Mediation analysis further indicated that rTMS reduced postoperative anxiety (β = ‐0.642, *p* = 0.048) and depression scores (β = ‐0.669, *p* = 0.021) (eTable S4, Figure 4D, E).

Additionally, rTMS was associated with improved postoperative frailty scores, with clearer differences on postoperative day 1 and day 7, whereas the day 3 difference was not robust after correction (Table 2). Frailty has been linked to an increased risk of POD, potentially mediated by inflammatory and neuroendocrine pathways (Cheng et al. 2024; Fu et al. 2022; Liu et al. 2022). rTMS may influence frailty through neuromodulatory and anti‐inflammatory effects, while improvements in pain, sleep, and psychological well‐being may further support early recovery and contribute to lower frailty status.

Notably, several secondary outcomes showed a time‐dependent pattern. Between‐group differences were more apparent on postoperative days 1 and 3, and were diminished by postoperative day 7 for some measures. This pattern suggests that the current brief, preoperative rTMS protocol may primarily influence early perioperative symptoms, including pain, sleep disturbance, and acute stress responses. In addition, standardized postoperative analgesia and routine recovery care may attenuate between‐group differences over time, and floor effects associated with symptoms improvement may further limit detectable separation by day 7. Future studies should examine whether extended treatment courses or postoperative reinforcement to achieve a cumulative effect, together with longer follow‐up, can produce more sustained improvements in these outcomes.

To examine whether the effects of rTMS on POD were mediated through improvements in pain, sleep, anxiety, or depression scores, we conducted a mediation analysis. The results indicated that the association between rTMS and POD was not explained by changes in these secondary outcomes (eTable S4, Figure 4B‐E). This finding suggests that rTMS may act through more direct neurobiological mechanisms.

In contrast, our study did not show evidence that rTMS affected PONV (Table 2). PONV is primarily associated with the administration of inhaled anesthetics and opioids (Horn et al. 2014). Its regulation involves the medulla oblongata vomiting center and related brainstem mechanisms (Horn et al. 2014), beyond the modulatory effects of prefrontal stimulation. Similarly, this study did not observe a significant reduction in postoperative CRP levels with rTMS, although a decreasing trend was noted (Table 2). This finding may not be fully consistent with the anti‐inflammatory effects of rTMS discussed above. After surgery and anesthesia, inflammatory cytokines typically increase and peak (Margraf et al. 2020; Patel et al. 2022), and because the intervention was delivered preoperatively, it may not have been sufficient to modulate this perioperative surge. Moreover, the limited number of rTMS sessions may have been insufficient to produce a cumulative anti‐inflammatory effect, which could explain the absence of a significant change.

The safety profile of rTMS was also evaluated in this trial (Table 3). All participants tolerated rTMS treatment, with no serious adverse events. The adverse events observed were transient and mild, including mild headache, scalp discomfort, muscle twitching, dizziness, and neck discomfort, all of which were reported as tolerable by participants. These symptoms typically resolved spontaneously within a short time after stimulation ceased. Such adverse reactions are attributed to direct scalp stimulation, stimulation‐induced muscle contractions, or the physical presence of the coil and headgear during treatment. Our findings are consistent with previous reports confirming the favorable safety profile of rTMS across different clinical populations (Fernandes et al. 2024; Ren et al. 2025).

This trial has several limitations. First, it was a single‐center trial with a relatively small sample size, which may limit the generalizability of the findings. Larger, multicenter randomized controlled trials are required to validate and extend these results. Second, sensory differences between active and sham rTMS may have compromised blinding and introduced expectancy bias. Because we did not formally assess blinding success (e.g., by asking participants or assessors to guess group assignment), expectancy bias cannot be fully excluded. Future studies using a dedicated sham coil and formal blinding assessment may further reduce this risk. Third, coil localization relied on the 10–20 system, which may be affected by interindividual anatomical variability and could lead to imprecise targeting of the left DLPFC; future studies should consider MRI‐guided neuronavigation or other coordinate‐based methods. Fourth, assessing POD only on postoperative days 1, 3, and 7 (twice daily at each time point) may have missed fluctuating delirium episodes and underestimated incidence, and outcomes beyond day 7 were not assessed. Missed assessments were not prospectively recorded, so the rate of missed assesssments could not be reported. Fifth, this trial did not integrate neuroimaging (e.g., fMRI, EEG) or biomarker analyses (e.g., BDNF, NfL, p‐tau) to investigate underlying mechanisms, which may provide deeper insights into its biological effects and neural correlates.

## 5 Conclusion

In this randomized controlled trial, preoperative HF‐rTMS targeting the left DLPFC was associated with a reduced risk of POD in elderly patients undergoing elective non‐cardiac surgery. These findings support the feasibility of perioperative rTMS as a potential non‐pharmacological preventive approach. However, broader application should await external validation and protocol optimization, particularly given the single‐center design, the brief stimulation course, and limitations related to delirium assessment and targeting precision. Future studies should confirm efficacy in multicenter trials, optimize stimulation parameters, extend follow‐up duration, incorporate neuronavigation‐based targeting, and compare rTMS with established delirium‐prevention strategies in pragmatic perioperative settings.

## Author Contributions

Wen‐Bo Gao, Wen‐Hui Wang: writing – original draft, visualization, formal analysis, data curation, conceptualization. Si‐Tong Zhou, Zi‐Wei Lu, Xin‐Xiang: investigation, data curation. Ting‐Yu Jin, Jin‐Hu: methodology, project administration. Chao‐Bo Ni: writing – review & editing, Conceptualization. Hua‐Dong Ni: writing – review & editing, project administration, methodology, conceptualization. Ming‐Yao: supervision, resources, project administration, funding acquisition, conceptualization.

## Funding

This study was supported by the National Natural Science Foundation of China (82171216), Natural Science Foundation of Zhejiang Province of China (LTGC23H090002), Science and Technology Project of Jiaxing City (2023AY31025), Zhejiang Multidisciplinary Innovation Team of Traditional Chinese Medicine for Diagnosis and Treatment of Elderly Headache and Vertigo (2022‐19), Zhejiang Clinovation Pride‐Herpes zoster neuralgia (CXTD202502014), Zhejiang Provincial Clinical Key Specialties‐Anesthesiology (2023‐ZJZK‐001), the Scientific Research Fund of National Health Commission‐Zhejiang Provincial Health Major Science and Technology Plan Project (WKJ‐ZJ‐2448), and Zhejiang Provincial Program of Traditional Chinese Medicine Science and Technology (2024ZL170).

## Conflicts of Interest

The authors declare no conflicts of interest.

## Institutional Review Board Statement

The study was conducted in accordance with the Declaration of Helsinki and approved by the Ethics Committee of the Affiliated Hospital of Jiaxing University (Ethics identifier: 2024‐LY‐794).

## Informed Consent Statement

Written informed consent has been obtained from all participants prior to enrollment in this study. Written informed consent has been obtained from the patients to publish this paper. The trial completed registration at the China Clinical Trial Registry (Identifier: CHiCTR2400094439).

## Supporting information




**Supplementary Material**: brb371242‐sup‐0001‐SuppMat.docx

## Data Availability

All original datasets are available on request to the corresponding author.
