# Supplementary material for: Efficacy of Repetitive Transcranial Magnetic Stimulation on Postoperative Delirium in Elderly Patients Undergoing Non‐Cardiac Major Surgery: A Randomized Controlled Trial
Source: Brain Behav. 2026 Jan 30;16(2):e71242. doi: 10.1002/brb3.71242 (PMC12856374; doi:10.1002/brb3.71242)
Supplement: Supplementary file 1 — Supplementary Material: brb371242‐sup‐0001‐SuppMat.docx [file BRB3-16-e71242-s001.docx]

**Supplementary Materials**

**eTable.1.** Baseline characteristics of study participants

**eTable.2.** Intraoperative and postoperative data among two groups

**eTable.3.** Categorized distributions of postoperative pain and PONV NRS scores by group.

**eTable.4.** Univariable and multivariate logistic regression analysis of the factors associated with postoperative delirium

**eTable.5.** Mediating Model Examination by Bootstrap

**eFig.1.** Forest plot of the subgroup analysis for the primary outcome

| **eTable.1.** Baseline characteristics of study participants | | | |
| --- | --- | --- | --- |
| Characteristic | Active-rTMS(n=122) | Sham-rTMS(n=123) | *P* value |
| Age, median (IQR), year | 69.0(63.0-73.0) | 68.0(63.0-73.0) | 0.973 |
| Sex, No. (%) |  |  | 0.567 |
| Male | 63(51.6) | 68(55.3) |  |
| Female | 59(48.4) | 55(44.7) |  |
| BMI, mean (SD), kg m^-2 | 23.4(3.1) | 23.8(3.4) | 0.309 |
| Education level, No. (%) |  |  | 0.482 |
| Illiteracy | 41(33.6) | 31(25.2) |  |
| Elementary school | 38(31.1) | 50(40.7) |  |
| Middle school | 30(24.6) | 28(22.8) |  |
| High school | 9(7.4) | 11(8.9) |  |
| College graduate | 4(2.9) | 3(2.4) |  |
| ASA classification, No. (%) |  |  | 0.241 |
| Ⅱ | 83(68.0) | 92(74.8) |  |
| Ⅲ | 39(32.0) | 31(25.2) |  |
| Type of operation, No. (%) |  |  | 0.140 |
| Abdominal surgery | 58(47.5) | 74(60.2) |  |
| Orthopedic surgery | 43(35.2) | 33(26.8) |  |
| Thoracic surgery | 21(17.2) | 16(13.0) |  |
| MMSE score, median (IQR) | 23.0(20.0-26.0) | 22.0(20.0-25.0) | 0.578 |
| SDRS score, median (IQR) | 5.0(5.0-7.0) | 6.0(5.0-7.0) | 0.238 |
| HADS-A score, median (IQR) | 2.0(0.0-2.3) | 2.0(0.0-2.0) | 0.312 |
| HADS-D score, median (IQR) | 0.0(0.0-1.0) | 0.0(0.0-1.0) | 0.631 |
| FRAIL scores, median (IQR) | 0.0(0.0-0.0) | 0.0(0.0-0.0) | 0.389 |
| Smoking, No. (%) | 30(24.6) | 26(21.1) | 0.520 |
| Drinking, No. (%) | 16(13.1) | 17(13.8) | 0.871 |
| Comorbidities, No. (%) |  |  | 0.389 |
| Hypertension | 60(49.2) | 67(54.5) |  |
| Diabetes | 21(17.2) | 22(17.9) |  |
| Cardiovascular diseases | 9(7.4) | 11(8.9) |  |
| Respiratory diseases | 7(5.7) | 3(2.4) |  |
| History of malignancy | 14(11.5) | 21(17.1) |  |
| TG, median (IQR), mmol/l | 1.5(1.1-2.2) | 1.5(1.1-2.2) | 0.557 |
| TC, mean (SD), mmol/l | 4.7(1.1) | 4.5(0.9) | 0.318 |
| LDL-C, mean (SD), mmol/l | 2.7(0.8) | 2.6(0.7) | 0.156 |
| HDL-C, median (IQR), mmol/l | 1.3(1.1-1.5) | 1.3(1.1-1.5) | 0.712 |
| **Abbreviations**: rTMS, repetitive transcranial magnetic stimulation; ASA, American Society of Anesthesiologists physical status classification; BMI, Body Mass Index (calculated as weight in kilograms divided by height in meters squared); MMSE, Mini-Mental State Examination score; SDRS, Sleep Dysfunction Rating Scale; HADS-A, Hospital Anxiety and Depression Scale - Anxiety; HADS-D, Hospital Anxiety and Depression Scale – Depression; FRAIL, FRAIL Scale; TG, Triglyceride; TC, Total Cholesterol; IQR, interquartile range; SD, standard deviation. | | | |

| **eTable.2.** Intraoperative and postoperative data among two groups. | | | | |
| --- | --- | --- | --- | --- |
| Characteristic | Active-rTMS(n=122) | | Sham-rTMS(n=123) | *P* value |
| **Intraoperative** | |  |  |  |
| Duration of surgery, median (IQR), min | | 155.0(125.0-194.8) | 152.0(125.0-200.0) | 0.898 |
| Duration of anesthesia, median (IQR), min | | 175.0(150.0-220.8) | 173.0(144.0-224.0) | 0.708 |
| Extubation time, median (IQR), min | | 42.5(25.0-93.5) | 41.0(25.0-95.0) | 0.952 |
| Infusion quantity, median (IQR), ml | | 1500.0(1000.0-2000.0) | 1500.0(1000.0-2000.0) | 0.179 |
| Estimated blood loss, median (IQR), ml | | 50.0(20.0-100.0) | 50.0(20.0-100.0) | 0.457 |
| **Postoperative** | |  |  |  |
| In-hospital delirium, No. (%) | | 10(8.2) | 35(28.5) | ＜0.001 |
| Worst delirium severity, mean (IQR) | | 21.5(20.5-23.3) | 19.0(18.0-22.0) | 0.141 |
| Type of delirium, No. (%) | |  |  | 0.434 |
| Hypoactive | | 8(6.6) | 27(22.0) |  |
| Hyperactive | | 0(0.0) | 3(2.4) |  |
| Mixed | | 2(1.6) | 5(4.1) |  |
| NRS score for Pain, median (IQR) | |  |  |  |
| T1 | | 2(2-3) | 3(2-3) | <0.001 |
| T3 | | 2(1-2) | 2(2-2) | <0.001 |
| T5 | | 0(0-1) | 0(0-1) | 0.018 |
| SDRS score, median (IQR) | |  |  |  |
| T1 | | 10(9-11) | 11(10-13) | <0.001 |
| T3 | | 8(7-9) | 9(8-11) | <0.001 |
| T5 | | 6(5-7) | 7(6-8) | 0.017 |
| NRS score for PONV, median (IQR) | |  |  |  |
| T1 | | 0(0-1) | 0(0-2) | 0.320 |
| T3 | | 0(0-0) | 0(0-0) | 0.207 |
| T5 | | 0(0-0) | 0(0-0) | >0.999 |
| FRAIL scores, median (IQR) | |  |  |  |
| T1 | | 2(2-3) | 3(2-3) | 0.001 |
| T3 | | 2(1-2) | 2(2-2) | 0.044 |
| T5 | | 1(0-1) | 1(1-2) | <0.001 |
| HADS-A score, median (IQR) | |  |  |  |
| T3 | | 2(2-3) | 3(2-3) | <0.001 |
| T5 | | 1(0-2) | 2(1-2) | <0.001 |
| HADS-D score, median (IQR) | |  |  |  |
| T3 | | 2(1-2) | 2(1-3) | <0.001 |
| T5 | | 0(0-0) | 0(0-1) | <0.001 |
| CRP, median (IQR), mg/L | | 32.4(15.2-53.5) | 37.2(21.5-59.4) | 0.101 |
| Duration of hospitalization, median (IQR), day | |  |  |  |
| Total | | 12.0(8.0-17.0) | 12.0(9.0-16.0) | 0.458 |
| After surgery | | 8.0(6.0-12.0) | 8.0(7.0-11.0) | 0.391 |

| *P* values are from two-sided Mann–Whitney U tests for between-group comparisons at each time point. For comparisons across three postoperative time points, statistical significance was interpreted using a Bonferroni-adjusted threshold of *p* < 0.017 (0.05/3).  **Abbreviations**: rTMS, repetitive transcranial magnetic stimulation; NRS, Numeric Rating Scale score; SDRS, Sleep Dysfunction Rating Scale; PONV, Postoperative Nausea and Vomiting; HADS-A, Hospital Anxiety and Depression Scale - Anxiety; HADS-D, Hospital Anxiety and Depression Scale – Depression; IQR, interquartile range; SD, standard deviation. |
| --- |

| **eTable.3. Categorized distributions of postoperative pain and PONV NRS scores by group.** | | | | | | |
| --- | --- | --- | --- | --- | --- | --- |
|  | Pain NRS at T3 | | Pain NRS at T5 | | PONV NRS at T3 | |
| Score | Active-rTMS (n= 124), No. (%) | Sham-rTMS (n= 125 ), No. (%) | Active-rTMS (n= 124), No. (%) | Sham-rTMS (n= 125 ), No. (%) | Active-rTMS (n= 124), No. (%) | Sham-rTMS (n= 125 ), No. (%) |
| 0 | 4(3.2) | 2(1.6) | 84(67.7) | 68(54.4) | 118(95.2) | 114(91.2) |
| 1 | 57(46.0) | 27(21.6) | 30(24.2) | 38(30.4) | 3(2.4) | 4(3.2) |
| ≥2 | 63(53.8) | 96(76.8) | 10(8.1) | 19(15.2) | 3(2.4) | 7(5.6) |
| Analyses are based on the ITT population.  Values are No (%), with percentages calculated within each treatment group; Categories: 0, 1, and ≥2.  **Abbreviations:** NRS, Numeric Rating Scale score; PONV, Postoperative Nausea and Vomiting; T3, postoperative days 3; T5, postoperative days 7. | | | | | | |

| **eTable.4.** Univariable and multivariate logistic regression analysis of the factors associated with postoperative delirium. | | | | | | | |
| --- | --- | --- | --- | --- | --- | --- | --- |
| Variables | Univariable logistic analysis | | | Multivariate logistic analysis | | | |
|  | OR | 95% CI | *P* value | aOR | 95% CI | *P* value | |
| rTMS | 0.22 | 0.10-0.46 | ＜0.001 | 0.15 | 0.06-0.36 | | ＜0.001 |
| Sex | 0.54 | 0.28-1.03 | 0.062 | 1.18 | 0.50-2.73 | | 0.706 |
| Age | 1.07 | 1.01-1.13 | 0.017 | 0.97 | 0.89-1.05 | | 0.416 |
| ASA | 2.73 | 1.41-5.29 | 0.003 | 2.22 | 0.92-5.38 | | 0.077 |
| BMI | 1.00 | 0.91-1.10 | 0.992 |  |  | |  |
| Education level | 0.15 | 0.02-1.10 | 0.061 | 1.92 | 0.18-20.68 | | 0.591 |
| MMSE socre | 0.61 | 0.50-0.73 | ＜0.001 | 0.57 | 0.44-0.73 | | ＜0.001 |
| Type of operation |  |  | 0.186 |  |  | |  |
| Abdominal surgery | 1.00 | Ref |  |  |  | |  |
| Orthopedic surgery | 0.65 | 0.31-1.36 |  |  |  | |  |
| Thoracic surgery | 0.40 | 0.13-1.20 |  |  |  | |  |
| Duration of anesthesia | 1.01 | 1.00-1.01 | 0.001 | 1.01 | 1.00-1.01 | | 0.080 |
| Extubation time | 1.01 | 1.00-1.01 | 0.001 | 1.00 | 1.00-1.01 | | 0.921 |
| Smoking | 0.76 | 0.35-1.70 | 0.509 |  |  | |  |
| Drinking | 0.73 | 0.27-2.01 | 0.544 |  |  | |  |
| TC | 0.91 | 0.66-1.24 | 0.537 |  |  | |  |
| TG | 0.93 | 0.68-1.27 | 0.646 |  |  | |  |
| HADS-A socre (T0) | 1.15 | 0.96-1.36 | 0.128 |  |  | |  |
| HADS-D socre (T0) | 1.02 | 0.65-1.60 | 0.931 |  |  | |  |
| SDRS socre (T0) | 1.11 | 0.96-1.29 | 0.149 |  |  | |  |
| FRAIL socre (T0) | 1.64 | 0.86-3.14 | 0.135 |  |  | |  |
| Specific subgroups: Sex (male vs. female), Education level (≤ 9 vs. > 9), Type of surgery (abdominal surgery vs. orthopedic surgery, abdominal surgery vs. thoracic surgery), preoperative Mini-Mental State Examination score (< 27 vs. ≥ 27), Smoking (yes vs. no) , Drinking (yes vs. no) ; T0: Surgery eve.  **Abbreviations:** OR, Odds Ratio; aOR, adjusted Odds Ratio; CI, Confidence Interval; Ref, Reference; TC, Total Cholesterol; TG, Triglyceride; SDRS, Sleep Dysfunction Rating Scale; HADS-A, Hospital Anxiety and Depression Scale - Anxiety; HADS-D, Hospital Anxiety and Depression Scale – Depression; FRAIL, FRAIL Scale. | | | | | | | |
|  | | | | | | | |

| **eTable.5.** Mediating model examination by bootstrap. | | | |
| --- | --- | --- | --- |
|  | Mediating effect | LL 95%CI | UL 95%CI |
| Pain | 0.048 | -0.832 | 0.524 |
| SDRS | -0.194 | -15.516 | 0.772 |
| Anxiety | -0.102 | -7.304 | 0.128 |
| Depression | -0.074 | -0.559 | 1.098 |
| **Abbreviations**: SDRS, Sleep Dysfunction Rating Scale; LL, Lower Limit; UL, Upper Limit. | | | |


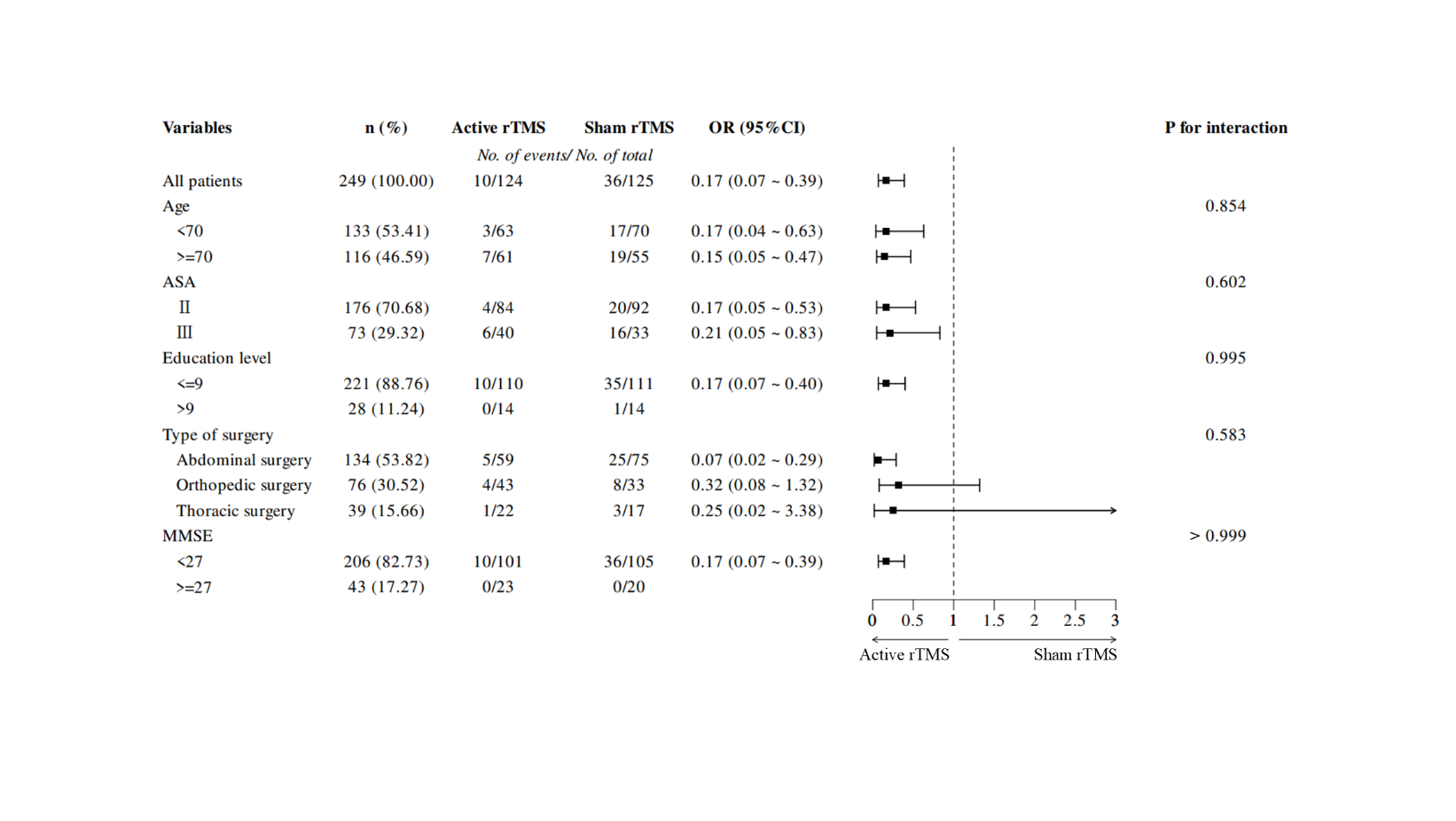
**eFig.1.** **Forest plot of the subgroup analysis for the primary outcome.**

Post-hoc exploratory subgroup analysis was performed based on age (< 70 vs. ≥ 70), ASA classification (Ⅱ vs. Ⅲ), education level (≤ 9 vs. > 9), type of surgery (abdominal surgery vs. orthopedic surgery, abdominal surgery vs. thoracic surgery), and preoperative Mini-Mental State Examination score (< 27 vs. ≥ 27).To determine the effect of the intervention in that particular subgroup, the effect of the intervention method (relative risk [95% CI (confidence interval)]) is presented separately in each subgroup. The interaction term is a test of whether the effect of the experimental intervention is statistically different in significance between subgroups.
